# Facile Fabrication of ZnO-ZnFe_2_O_4_ Hollow Nanostructure by a One-Needle Syringe Electrospinning Method for a High-Selective H_2_S Gas Sensor

**DOI:** 10.3390/ma15020399

**Published:** 2022-01-06

**Authors:** Kee-Ryung Park, Ryun Na Kim, Yoseb Song, Jinhyeong Kwon, Hyeunseok Choi

**Affiliations:** 1 Smart Manufacturing System R&D Department, Korea Institute of Industrial Technology (KITECH), 89 Yangdaegiro-gil, Ipjang-myeon, Seobuk-gu, Chungchengnam-do, Cheonan 31056, Korea; pkr86@kitech.re.kr; 2 Department of Energy Engineering, Dankook University, 119, Dandae-ro, Dongnam-gu, Chungcheongnam-do, Cheonan-si 31116, Korea; imryunna@naver.com; 3 Korea Institute for Rare Metals, Korea Institute of Industrial Technology (KITECH), 156 Gaetbeol-ro, Yeonsu-gu, Incheon 21999, Korea; songys88@kitech.re.kr

**Keywords:** ZnO, ZnFe_2_O_4_, hollow nanostructure, electrospinning, H_2_S, gas sensor

## Abstract

Herein, a facile fabrication process of ZnO-ZnFe_2_O_4_ hollow nanofibers through one-needle syringe electrospinning and the following calcination process is presented. The various compositions of the ZnO-ZnFe_2_O_4_ nanofibers are simply created by controlling the metal precursor ratios of Zn and Fe. Moreover, the different diffusion rates of the metal oxides and metal precursors generate a hollow nanostructure during calcination. The hollow structure of the ZnO-ZnFe_2_O_4_ enables an enlarged surface area and increased gas sensing sites. In addition, the interface of ZnO and ZnFe_2_O_4_ forms a p-n junction to improve gas response and to lower operation temperature. The optimized ZnO-ZnFe_2_O_4_ has shown good H_2_S gas sensing properties of 84.5 (S = R_a_/R_g_) at 10 ppm at 250 °C with excellent selectivity. This study shows the good potential of p-n junction ZnO-ZnFe_2_O_4_ on H_2_S detection and affords a promising sensor design for a high-performance gas sensor.

## 1. Introduction

The development of IoT-based smart sensor systems including smart devices [1], self-driving cars [2], home automation [3], and data mining [4] has been intensively prompted to evolve various environmental sensors. Particularly, semiconductor-based gas sensors have been broadly studied due to clear working principles, simple device structure, and good response activity to toxic gases [5,6]. Typical semiconductor gas sensors are manufactured via complex stepwise photolithography processes [7,8]. Although the traditional fabrication process ensures massive wafer-scale productivity, this method cannot meet small-volume and on-demand capacities. In addition, fragile supply chain issues on the process materials lead to increasing total production time and cost. Therefore, the demand for facile fabrication routes with common materials is increasing.

Hydrogen sulfide (H_2_S) is one of the hazardous gas molecules of which even tiny concentrations bring permanent damages to humans’ metabolic and nervous systems [9]. However, it is derived from commonly accessible environments such as rotten foods, water purification plants, and underground sewer networks. Therefore, a H_2_S monitoring sensor with a fast response time and low operation temperature is required to prevent gas poisoning. Various semiconductor metal oxide nanomaterials including CuO [10], Fe_2_O_3_ [11], In_2_O_3_ [12], SnO_2_ [13], WO_3_ [14], and ZnO [15] have been explored for H_2_S gas sensor applications. While ZnO has been widely used as a gas sensor application due to its properties of non-toxic, low cost, excellent physical/chemical stability, and n-type wide bandgap of 3.2 eV, the broad/unconfined gas sensing characteristics of the ZnO nanostructure easily tends to lose selectivity for the target gas [16].

To enhance electrical properties and gas sensitivity, the intact ZnO nanostructure is manipulated to get heterojunction nanostructure by adding complex metal oxides including spinel and/or perovskite [17]. ZnFe_2_O_4_ is a spinel-structured ferrite with a narrow bandgap (−1.9 eV) semiconductor material with high surface activity and low activation energy. Owing to the low activation energy, the ZnFe_2_O_4_ is capable of detecting reducing gases with high response signals [18]. Therefore, the ZnO-ZnFe_2_O_4_ heterojunction structure has anticipated obtaining enhanced gas sensing properties by adjusting the Fermi energy level, electron depletion region, and potential barrier at its interface. Several efforts have been dedicated to making a heterojunction nanostructure of ZnO-ZnFe_2_O_4_ in previous studies. For example, Wang et al. reported ZnO-ZnFe_2_O_4_ composite hollow microspheres via the hydrothermal method [19]. The fabricated ZnO-ZnFe_2_O_4_ hollow microspheres have been adopted as a volatile organic pollutant detectable sensor for n-butanol, acetone, ethanol, and methanol. The ZnO-ZnFe_2_O_4_ composite hollow microspheres-based gas sensor has shown high response, good reversibility, and fast on/off characteristics during operation temperatures of as high as 320 °C. Similarly, Zhang et al. executed the synthesis of hollow ZnFe_2_O_4_-ZnO hybrid spheres with good uniformity and high crystalline structure by using a two-step synthetic method [20]. The hybrid spheres turned into a paste-type solution and were coated on the alumina tube by dipping. The acetone gas-sensing performance was recorded at sensing concentrations of 10 to 200 ppm at 280 °C. Although these studies reported meaningful gas sensing performances, several issues have been revealed such as material synthetic yield and high operating temperature.

In this study, the ZnO-ZnFe_2_O_4_ hollow nanofibers were fabricated by the electrospinning process. The hollow nanofiber structure has advantages of high surface-to-volume area for inner/outer structure, high porosity, and good chemical/mechanical stability [21,22]. Furthermore, the fabrication route provides facile nanomaterial manipulations, high productivity, and controlled stable outputs from the homogeneous mixture of the metal precursor solution. In particular, the ZnO-ZnFe_2_O_4_ nanofibers were acquired by using a one-needle syringe condition. Various ratios of Zn and Fe precursors were mixed as a processing solution to make different compositions of ZnO-ZnFe_2_O_4_ nanofibers. The various as-spun ZnO-ZnFe_2_O_4_ nanofibers were calcined at 600 °C for 3 h to remove residues and to convert to a high porosity nanostructure. After the calcination process, the ZnO-ZnFe_2_O_4_ nanofibers became porous and hollow structures, having enhanced surface area. The fabricated ZnO-ZnFe_2_O_4_ hollow nanofibers have been employed as a high-sensitive gas sensor application for detecting H_2_S gas.

## 2. Experimental Section

### 2.1. Material Preparations

Zn(NO_3_)_3_∙6H_2_O, Fe(NO_3_)_3_∙9H_2_O, ethanol, N,N-dimethylformamide (DMF), and poly(vinylpyrrolidene) (PVP; Mw = 1,300,000) were purchased from Sigma-Aldrich, USA. All chemicals were used as received without further purification.

### 2.2. Preparations of ZnO-ZnFe_2_O_4_ Hollow Nanofibers

ZnO-ZnFe_2_O_4_ hollow nanofibers were formed via an electrospinning process including calcination at 600 °C by the Kirkendall effect. To prepare the electrospinning solution, one-dimensional hollow nanofibers containing Zn and Fe precursors and PVP were synthesized via a single-nozzle electrospinning technique. To prepare the solution, 0.5 g Zn(NO_3_)_3_∙6H_2_O and 20 wt% Fe(NO_3_)_3_∙9H_2_O were dissolved in a mixed solution of ethanol and DMF (wt% = 1:1) with 1 g of DI water under stirring at room temperature for 1 h. Then, 0.45 g of PVP was added to the dissolved solution of Zn and Fe precursors. The resulting solution was loaded into a syringe for electrospinning at a flow rate of 0.2 mL/h under an applied voltage of 19 kV with 15 cm of the distance of the needle-tip-collector. The as-spun nanofibers were calcinated at 600 °C with increasing temperature rate of 3 °C/min for 3 h. Finally, ZnO-ZnFe_2_O_4_ hollow nanofibers were obtained.

### 2.3. Material Characterizations

The synthesized materials were characterized using scanning electron microscopy (SEM, S-4800, Hitachi High-Technologies, Co., Tokyo, Japan), X-ray diffractometry (XRD, D/MAX-2500-PC, Rigaku International Co., Tokyo, Japan) with Cu Kα X-ray source (λ = 1.5418 Å) at 2θ, high resolution transmission electron microscopy (HR-TEM, JEM-2100 F, JEOL Ltd., Tokyo, Japan), high-angle annular dark-field scanning tunneling electron microscopy (HAADF-STEM), selected-area electron diffraction (SAED), energy dispersive X-ray spectroscopy (EDS), and X-ray photoelectron spectroscopy (Theta Probe XPS, ThermoFisher Scientific, Waltham, MA, USA) with a base pressure of 4.8 × 10^−9^ mbar using a monochromatic Al Kα X-ray source (hυ = 1486.6 eV)

### 2.4. Gas Sensor Performance Measurement

First, 200 mg of ZnO-ZnFe_2_O_4_ nanotubes were dispersed in 1000 μL of ethanol. The 3 μL of ZnO-ZnFe_2_O_4_ solution was then dropped on the SiO_2_ substrate with Au electrodes on a hot-plate at 60 °C for 15 min. The gas sensing tests were carried out using a company (GMC 1200, ATOVAC, Yongin, Korea) gas sensor measurement system equipped with a data acquisition system (2450 Sourcemeter, Keithley, Solon, OH, USA). The resistance was calculated by the recorded current from applied constant DC voltage of 5 V. The gas concentration was controlled by changing the mixing ratio of nitrogen and H_2_S (10 ppm in nitrogen) with fixed oxygen of 21% using mass flow controller (Model 5850E, Brooks Instrument, Hatfield, PA, USA) between 0.3 and 10 ppm. Nitrogen and oxygen were used as the carrier gas at a fixed flow rate of 500 sccm. The operating temperature was controlled from 100 to 280 °C using a ceramic heater. A schematic of the gas sensor system is illustrated in Supplementary Figure S1. Various analyte gases (CH_3_COCH_3_, NO_2_, and C_2_H_5_OH) were exposed to envisage the gas selectivity of the sensor as a function of different concentration. The response was used to characterize the sensor performance using the equation Response = R_a_/R_g_, where R_a_ and R_g_ are the electrical resistance of the sensor under dry air and the concentration of H_2_S gas, respectively.

## 3. Results

### 3.1. Structure and Surface Morphologies

**Figure 1:**
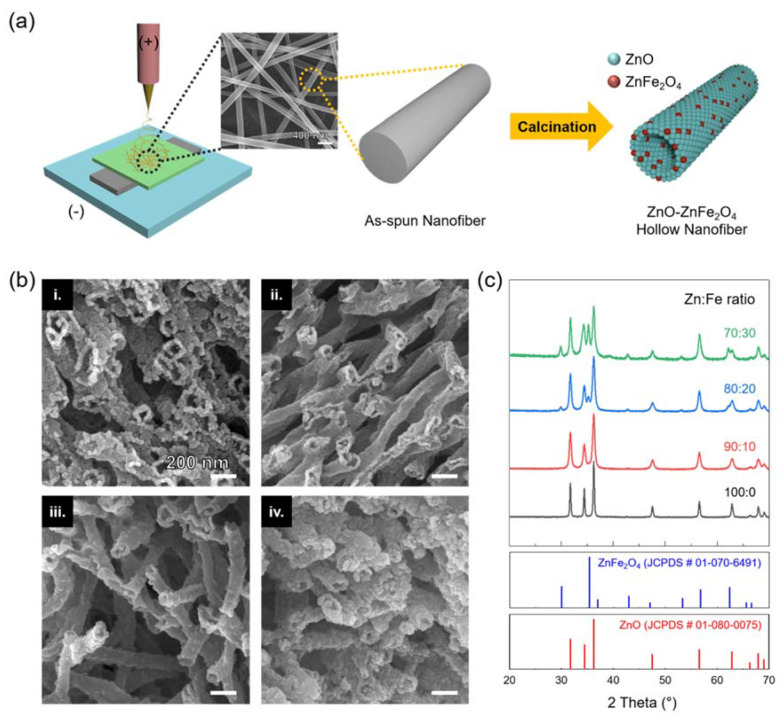
(**a**) Schematic process of the ZnO-ZnFe_2_O_4_ hollow nanofibers through one-needle syringe electrospinning and the following calcination process. (**b**) FE-SEM images of the ZnO-ZnFe_2_O_4_ hollow nanofibers of various compositions by controlling the precursor ratios of Zn and Fe for (i) 100:00, (ii) 90:10, (iii) 80:20, and (iv) 70:30. (**c**) XRD patterns of various ZnO-ZnFe_2_O_4_ hollow nanofibers.

The ZnO-ZnFe_2_O_4_ hollow nanofibers were fabricated by electrospinning and the following calcination process. In order to find the optimum synthetic compositions of the ZnO-ZnFe_2_O_4_ nanofibers, different Zn and Fe precursor ratios of 100:0, 90:10, 80:20, and 70:30 were used for the electrospinning process. As depicted in Figure 1a, the various composition of Zn and Fe precursors were dissolved in a mixed solution of PVP, ethanol, DMF, and deionized water for the electrospinning. The calcination process completely removed the unnecessary materials and formed hollow nanostructures for the various ratios of ZnO-ZnFe_2_O_4_ nanofibers. Furthermore, the metal precursor ingredients of zinc nitrate and iron nitrate converted towards ZnO and ZnFe_2_O_4_, respectively. At that moment, the hollow nanostructure was generated by the Kirkendall effect [13,23,24]. In detail, during the calcination process, the positions of the metal atoms are exchanged with the vacancies generated from the PVP removal. While the Zn and Fe ions rapidly diffuse to the outer surface of the nanofiber, the metal oxides are rarely spread out to the inner structure of the nanofiber. Accordingly, the different diffusion rates between the metal oxides and metal precursors make hollow nanostructures. Figure 1b represents various morphologies of the as-fabricated ZnO-ZnFe_2_O_4_ hollow nanofibers (i) 100: 0, (ii) 90:10, (iii) 80:20, and (iv) 70:30 wt%. All of the ZnO-ZnFe_2_O_4_ nanofibers had porous nanostructure, indicating enlarged surface area. Especially, the ratio of 80:20 result shows long-shapes of nanotube structure with approximately 150 nm in diameter with around 15 nm of wall thickness such that it provides relatively easy manipulation features. The XRD patterns of the fabricated various ZnO-ZnFe_2_O_4_ hollow nanostructure were compared by the Zn and Fe precursor ratios of 100:0, 90:10, 80:20, and 70:30 wt% as shown in Figure 1c. All the diffraction peaks in the spectrum correspond to the specific crystal planes of the hexagonal ZnO (JCPDS Card No. 01-080-0075) and the spinel ZnFe_2_O_4_ (JCPDS Card No. 01-070-6491), indicating that the ZnO-ZnFe_2_O_4_ nanocomposites were properly fabricated. No peaks were observed associated with other impurity phases. In addition, the increase of Fe content reduced the diffraction peaks intensity of ZnO and increased the intensity of ZnFe_2_O_4_ diffraction peaks in the materials.

### 3.2. TEM and XPS Analysis

**Figure 2:**
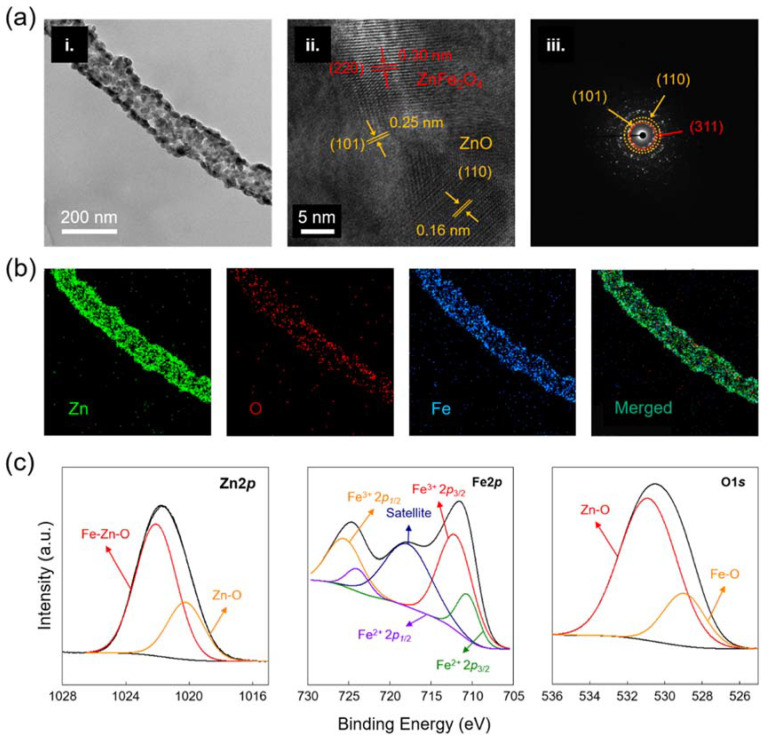
(**a**) TEM, HR-TEM, and SAED analysis results of the ZnO-ZnFe_2_O_4_ hollow nanofiber after calcination at 600 °C. (**b**) EDS mapping images of the ZnO-ZnFe_2_O_4_ hollow nanofiber. (**c**) XPS spectra of ZnO-ZnFe_2_O_4_ hollow nanofiber for Zn2p, Fe2p, and O1s.

The fabricated ZnO-ZnFe_2_O_4_ hollow nanostructure was further analyzed by TEM and EDS mapping. As mentioned, the ZnO-ZnFe_2_O_4_ hollow nanostructure from 80:20 wt% of Zn and Fe precursor ratio has a proper nanostructure, therefore, it was selected as a standard specimen in this study. The morphology images of the hollow shape and porous surface are observed in Figure 2a(i). The thickness of the individual nanotube was approximately 15 nm. The lattice distances of the ZnO and ZnFe_2_O_4_ are measured in Figure 2a(ii). For the ZnO, lattice distances on the (101) and (110) planes were observed. ZnFe_2_O_4_ showed an interplanar spacing of 0.30 nm for the (311) plane. Figure 2a(iii) indicates the SAED patterns of the ZnO-ZnFe_2_O_4_ hollow nanostructure, showing the fabricated nanostructures had polycrystalline and slight ring patterns for ZnO and ZnFe_2_O_4_. The EDS mapping images in Figure 2b represent homogeneous element distributions including Zn, O, and Fe for the ZnO-ZnFe_2_O_4_ hollow nanofiber. The chemical states of the fabricated ZnO-ZnFe_2_O_4_ hollow nanofiber were identified using XPS analysis, as shown in Figure 2c. The XPS examined three elements, Zn, Fe, and O. While the pristine ZnO had a significant peak at 1021 eV for Zn2p_3/2_, the measured Zn2p peak slightly shifted to around 1021.7 eV due to the existence of the Fe-containing component at 1022.1 Ev [25]. The Fe2p peaks consisted of different electron coordination of Fe^2+^ and Fe^3+^. ZnFe_2_O_4_ is a spinel structure material, so tetrahedral symmetry governs the electron state, therefore, signals of the Fe^3+^ peaks were stronger than those of the Fe^2+^ peaks [26,27]. Similarly, the O1s peak was formed with two major peaks of 530 and 531 eV, which is consistent with a value between ZnO and ZnFe_2_O_4_. Particularly, the low binding energy of 530 eV originated from the lattice O^2−^ ions and the high binding energy of 531 eV was consistent with adsorbed O^−^ and O_2_^−^ [25,28]. The morphology structures of porosity and tube shape play a crucial role in gas sensing performance. That is, the target gas can be easily diffused into/out towards the designed material so that the gas sensing property is increased and the response/recovery time is reduced.

### 3.3. Gas Sensing Performances

**Figure 3:**
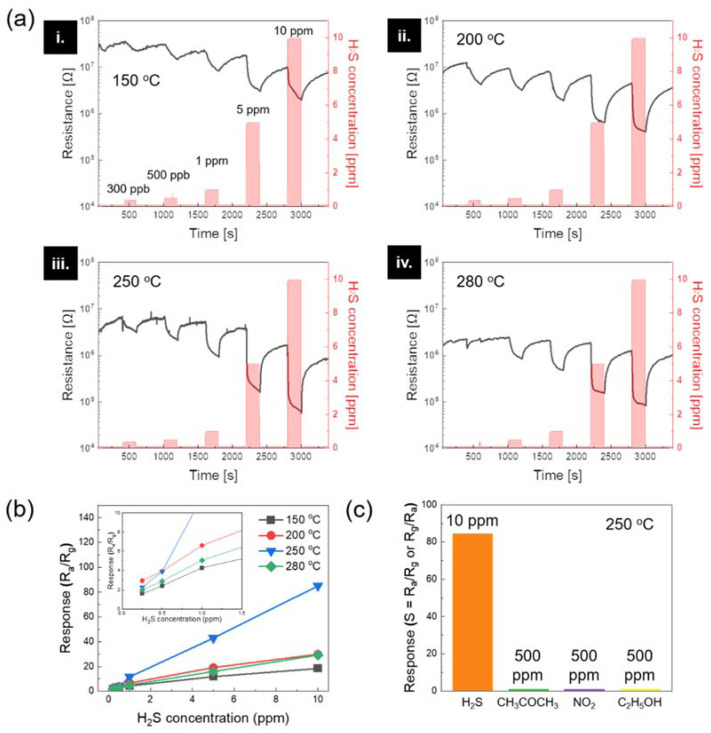
(**a**) H_2_S gas sensing performances of the ZnO-ZnFe_2_O_4_ hollow nanofibers with different gas concentrations from 300 ppb to 10 ppm with different operating temperatures of 150, 200, 250, and 280 °C. (**b**) Response-concentration fitting curves regarding operation temperature conditions. (**c**) Gas selectivity comparisons for the ZnO-ZnFe_2_O_4_ hollow nanofibers at 250 °C.

The fabricated ZnO-ZnFe_2_O_4_ hollow nanofibers were used for gas sensor applications. In order to find the optimal sensing composition and performance, the ZnO-ZnFe_2_O_4_ hollow nanofibers synthesized from various content ratios of Zn and Fe precursors from 100:0 to 70:30 wt% were evaluated regarding sensing characteristics of H_2_S gas at 250 °C (Supplementary Figure S2). As the Fe contents increased from 0 to 30 wt%, the initial resistance value in the air was also elevated due to the electron depletion region from the p-n junction. The fabricated ZnO-ZnFe_2_O_4_ hollow nanofiber from the ratio of 80:20 wt% showed better sensing properties than others. (Table S1) Therefore, it was selected as a standard sensing material for the H_2_S gas sensor. As shown in Figure 3a, the gas sensing properties of the ZnO-ZnFe_2_O_4_ hollow nanofiber were examined over a wide range of temperatures from 150 to 280 °C with different concentrations of H_2_S from 300 ppb to 10 ppm. Typically, for the semiconductor-type gas sensors, the chemical interaction between the sensor material and the target gas plays a significant role in the sensor reaction, and the temperature condition largely affects the chemical reaction: the low-temperature condition results in relatively low reaction signals since the oxygen species on the surface of the active materials have low thermal energy to interact. When the surrounding temperature rises, the interactions between an active material and gas increase for the following reasons: first, gas molecules with elevated thermal energy are able to overcome the energy barrier for the surface reactions. Second, oxygen species such as O_2_^−^ are generated on the surface of the active material. The generated oxygen species will react with more electrons from the surface of the active material [29]. On the other hand, sensitivity is dramatically reduced in the excessive high-temperature environment because the low gas adsorption capacity of gas molecules causes low utilization of the active material [30]. Accordingly, the response time declined and the sensing response increased when the temperature rises from 150 to 250 °C. Then, the sensing responses abruptly decreased when the operating temperature was over 250 °C. Supplementary Figure S3 shows a detailed analysis of the ZnO-ZnFe_2_O_4_ (20:80) hollow nanofiber sensor at 10 ppm and 280 °C. Electrical resistance was measured at around 10^6^ Ω at the initial stage in the air. Afterward, the target H_2_S gas was injected, the resistance rapidly decreased to 10^4^ Ω within 200 s. The response time (T_90_) was only 11 s. When the target gas injection was suspended, the electrical resistance was gradually recovered. The measured recovery time (D_10_) was 280 s. Owing to the hollow nanostructure, the reaction and recovery time have a trade-off relationship. The hollow nanostructure provides large surface areas and fast air/gas penetrations to the inside of the structure. At the same time, the enlarged surface prevents the target gas from being sent out of the hollow structure. The highest and minimum response signals (S = R_a_/R_g_) for H_2_S gas were individually detected at 10 ppm with 84.5 and 300 ppb with 2.27 when the temperature condition was 250 °C in Figure 3b. This sensitivity result is comparable and shows a superior sensing property over other ZnFe_2_O_4_-based H_2_S gas sensors as shown in Table S2. As depicts in Figure 3c, the selectivity of the ZnO-ZnFe_2_O_4_ hollow nanofiber sensor was executed towards various gases such as H_2_S, CH_3_COCH_3_, NO_2_, and C_2_H_5_OH at the operating temperature of 250 °C. The sensor responses were obtained as 84.5, 1.01, 1.01, and 1.05 for H_2_S (10 ppm), CH_3_COCH_3_ (500 ppm), NO_2_ (500 ppm), and C_2_H_5_OH (500 ppm), respectively. The ZnO-ZnFe_2_O_4_ hollow nanofiber showed very high selectivity to the H_2_S than other gases.

### 3.4. Mechanism

**Figure 4:**
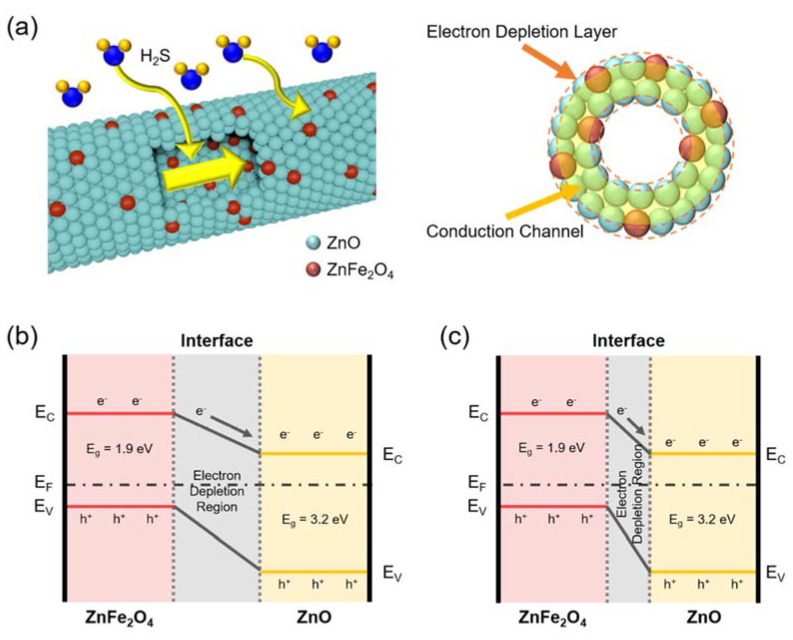
(**a**) Schematic of mechanism of the ZnO-ZnFe_2_O_4_ hollow nanofiber-based gas sensor. The hollow nanostructure provides enlarged surface areas and a diminished depletion layer. Band diagram of the ZnO-ZnFe_2_O_4_ heterostructure in (**b**) air and (**c**) H_2_S environment.

The H_2_S gas sensing mechanism of the ZnO-ZnFe_2_O_4_ hollow nanofiber is illustrated in Figure 4. The interface of the ZnO-ZnFe_2_O_4_ is similar to a p-n junction. Owing to the ZnO-ZnFe_2_O_4_ having a hollow nanostructure, H_2_S gas can react to the inner/outer sites of the nanofiber. When the H_2_S molecules react with the ZnO-ZnFe_2_O_4_ p-n junction, a chemical reaction occurs as follows: 2H_2_S (g) + 3O^−^ (ads) → 2SO_2_ + 2H_2_O + 3e^−^. Then, the generated electron flows to the active material and reduces the electrical resistance of the gas sensor. As the hollow nanotube structure has large surface areas, it is able to react with more gas sources. Therefore, the gas sensing characteristics are improved compared to the pristine ZnO nanotube structure by matching the appropriate p-n junction ratio. In other words, a typical p-n junction is formed at the interfaces when ZnFe_2_O_4_ nanoparticles meet ZnO. The electrons tend to diffuse from the ZnO to the ZnFe_2_O_4_. At the same time, a built-in electric field will be created at the interface to balance the diffusion motion [31]. Then, the interfacial electrons and holes are pulled to the opposite direction by the formed build-in electric field. This effect can minimize the electron-hole recombination at the interface, lead to effective separation of the charged carriers, and increase the free electron density [32]. Since more electrons are participating in the absorption and dissociation process of oxygen molecules, oxygen species are much increased on the surface of the ZnO-ZnFe_2_O_4_ composites compared to that of the pristine ZnO. Similarly, the thicker depletion layer is formed at the interface and it provides more sensitive properties to the target gas molecules for the ZnO-ZnFe_2_O_4_ hollow nanostructure. Therefore, when the ZnO-ZnFe_2_O_4_ gas sensor is exposed to H_2_S, more electrons are released back to the conduction band of the ZnO-ZnFe_2_O_4_, and thus a large conductance change can be achieved.

## 4. Conclusions

ZnO-ZnFe_2_O_4_ hollow nanofibers were fabricated by simple one-needle electrospinning with different ratios of Fe and Zn precursors. The as-spun nanofiber was calcined to remove unnecessary ingredients as well as to form a hollow nanostructure by different diffusion rates of metal oxides and metal precursors. The Zn and Fe precursor ratio of 80:20 verified the optimized surface structure and chemical properties through XRD, TEM, and XPS analysis. The diameter of the nanotube was 150 nm and the thickness of the shell was 15 nm. The H_2_S gas sensing characteristic was carried out with concentrations of 300 ppb to 10 ppm at the conditions of 150 and 280 °C, respectively. The interfaces of the ZnO-ZnFe_2_O_4_ generated a p-n junction which helped to obtain increased gas sensing properties. The ZnO-ZnFe_2_O_4_ gas sensing performance was recorded as 84.5 (S = R_a_/R_g_) at 250 °C at 10 ppm of H_2_S. The fabricated ZnO-ZnFe_2_O_4_ hollow nanofiber represented high sensitivity and excellent selectivity for the H_2_S gas application. Therefore, ZnO-ZnFe_2_O_4_ hollow nanofibers are good candidates for oxide-based gas sensors along with large surface areas as well as proper properties of the p-n junction.

## Data Availability

The data presented in this study are available on request from the corresponding author.

## Supplementary Materials

The following supporting information can be downloaded at: https://www.mdpi.com/article/10.3390/ma15020399/s1, Figure S1: A schematic of the gas sensor measurement system. Figure S2: Gas-sensing characteristics of ZnO-ZnFe_2_O_4_ nanotubes with different precursor ratio with Zn and Fe, Figure S3: Response and recovery properties of ZnO-ZnFe_2_O_4_ hollow nanofiber under 10 ppm H_2_S gas at 280 °C, Table S1: Selectivity data of the gas sensors fabricated with different ratios of Zn and Fe at conditions of 10 ppm of H_2_S gas and 250 °C. Table S2: Comparisons of the ZnFe_2_O_4_ nanostructure-based H_2_S gas sensors.
