# Supplementary material for: Facile Fabrication of ZnO-ZnFe2O4 Hollow Nanostructure by a One-Needle Syringe Electrospinning Method for a High-Selective H2S Gas Sensor
Source: Materials (Basel). 2022 Jan 6;15(2):399. doi: 10.3390/ma15020399 (PMC8782020; doi:10.3390/ma15020399)
Supplement: Supplementary file 1 [file materials-15-00399-s001.zip › materials-1500716-SI.pdf]

materials

MDPI

Facile Fabrication of ZnO-$ZnFe_2O_4$ Hollow Nanostructure by a One-Needle Syringe Electrospinning Method for a High-Selective H_2S Gas Sensor

Kee-Ryung Park[1], Ryun Na Kim[2], Yoseb Song[3], Jinhyeong Kwon[1]* and Hyeunseok Choi[1]*

[1] Smart Manufacturing System R&D Department, Korea Institute of Industrial Technology (KITECH), 89 Yang-daegiro-gil, Ipjang-myeon, Seobuk-gu, Chungchengnam-do, Cheonan, 31056, Korea
[2] Department of Energy Engineering, Dankook University, 119, Dandae-ro, Dongnam-gu, Cheonan-si, Chung-cheongnam-do, 31116, Korea
[3] Korea Institute for Rare Metals, Korea Institute of Industrial Technology, 21999, Incheon, Korea
* Correspondence: to jhs0909k@kitech.re.kr (J.K.); hchoi@kitech.re.kr (H.C.)

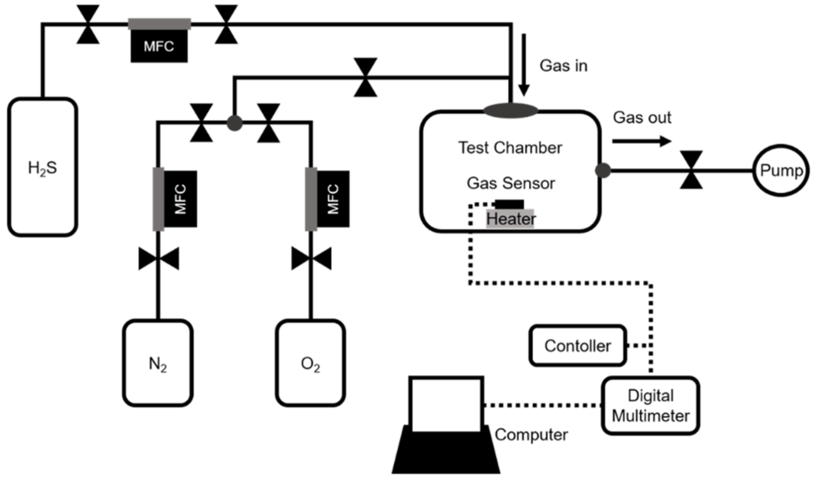

Figure S1. A schematic of the gas sensor measurement system.

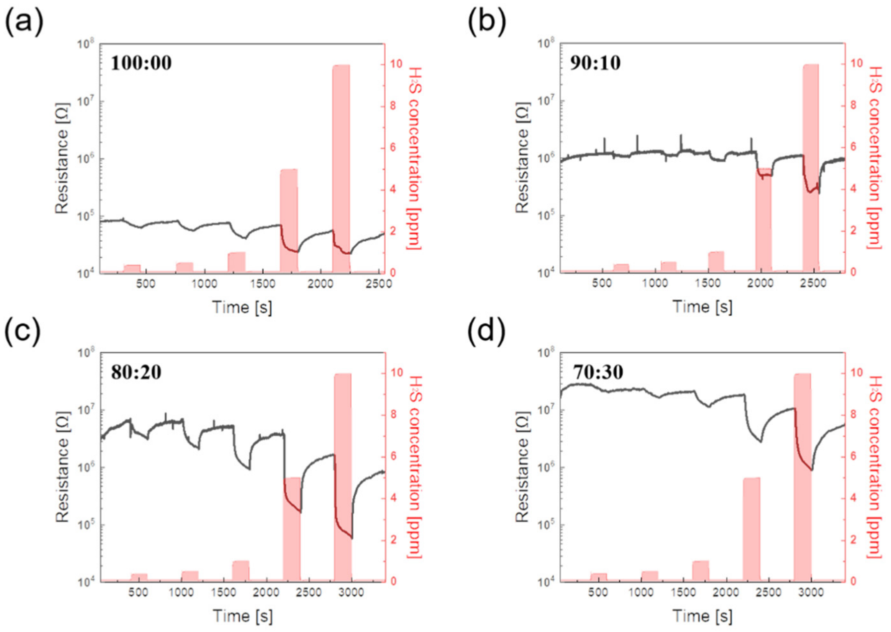

Figure S2. Gas sensing characteristics of ZnO-$ZnFe_2O_4$ nanotubes with different precursor ratio with Zn and Fe; (a) 100:00, (b) 90:10, (c) 80:20, and (d) 70:30 to H_2S gas in the range of 300 ppb – 10 ppm at 250 ^{o}C.

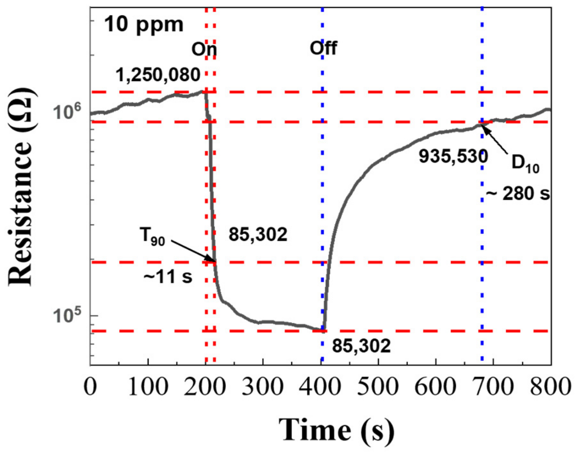

Figure S3. Response and recovery properties of the ZnO-$ZnFe_2O_4$ hollow nanofiber under 10 ppm H_2S gas at 280 °C.

Table S1. Selectivity data of the gas sensors fabricated with different ratios of Zn and Fe at conditions of 10 ppm of H_2S gas and 250 °C.

Ratios of Zn and Fe Precursors	Response ($S = R_a/R_g$)
100:0	4.05
90:10	4.91
80:20	84.5
70:30	30.95

Table S2. Comparisons of the $ZnFe_2O_4$ nanostructure-based H_2S gas sensors.

#	Types of the $ZnFe_2O_4$ nanostructures	Concentration (ppm)	Operating Temperature (°C)	Response ($S = R_g/R_a$)	Reference
1	$ZnFe_2O_4$ nanoparticle	10	135	23.3	[1]
2	Cu-doped $ZnFe_2O_4$ nanoparticle	10	100	18	[2]
3	Au-doped ZnFe2O4 micro-spheres	200	25	65.9	[3]
4	ZnFe2O4 nanoparticle	100	260	64	[4]
5	ZnO-ZnFe2O4 hollow nanostructure	10	250	84.5	This Work

References

1. H. Zhang, F. Meng, L. Liu, Y. Chen, P. Wang, "Highly sensitive H_2S sensor based on solvothermally prepared spinel $ZnFe_2O_4$ nanoparticles", *Journal of Alloys and Compounds*, 764, 147-154, 2018.
2. W. Zhang, Y. Shen, J. Zhang, H. Bi, S. Zhao, P. Zhou, C. Han, D. Wei, N. Cheng, "Low-temperature H_2S sensing performance of Cu-doped $ZnFe_2O_4$ nanoparticles with spinel structure", *Applied Surface Science*, 470, 581-590, 2019.
3. Y. Yan, P. Nizamidin, G. Turdi, N. Kari, A. Yimit, "Room-temperature H_2S Gas Sensor Based on Au-doped $ZnFe_2O_4$ Yolk-shell Microspheres", *Analytical Sciences*, 33, 945-951, 2017.
4. J. Wu, D. Gao, T. Sun, J. Bi, Y. Zhao, Z. Ning, G. Fan, Z. Xie, "Highly selective gas sensing properties of partially inversed spinel zinc ferrite towards H_2S", *Sensors and Actuators B: Chemical*, 235, 258-262, 2016.
